# Improvement in proteinuria with sodium-glucose cotransporter 2 inhibitors and esaxerenone treatment in patients with chronic allograft kidney disease: A case report 

**DOI:** 10.5414/CNCS111078

**Published:** 2024-03-15

**Authors:** Shoichiro Daimon

**Keywords:** sodium glucose cotransporter 2 inhibitor, mineralocorticoid receptor blocker, esaxerenone, proteinuria, kidney transplantation

## Abstract

Proteinuria is a predictor of end-stage renal disease. The effectiveness of an angiotensin-converting enzyme inhibitor or an angiotensin II receptor blocker for the reduction in urinary protein excretion and renoprotection in proteinuric chronic kidney disease patients is well known, and coadministration of and sodium-glucose cotransporter inhibitor and the mineralocorticoid receptor blocker eplerenone has recently demonstrated an additive albuminuria-lowering effect in chronic kidney disease patients. Proteinuria is also an independent predictor of end-stage renal disease in kidney transplant recipients. Sodium-glucose cotransporter 2 inhibitors were administered to a 60-year-old man with chronic allograft kidney disease who had increasing urinary protein excretion with valsartan treatment. Although urinary protein excretion decreased drastically, it later increased to the same levels. A nonsteroidal mineralocorticoid receptor blocker, esaxerenone, was added to these medications, again resulting in a decrease in urinary protein excretion. Although the long-term renoprotective effect is not known, these medicines are promising and safe agents to reduce urinary protein excretion in patients with chronic allograft kidney disease.

## Introduction 

Proteinuria is common in chronic kidney disease (CKD) patients and is an independent predictor of end-stage renal disease (ESRD) [1], and a reduction in albuminuria in CKD patients is associated with a reduced risk of ESRD [2]. Angiotensin-converting enzyme inhibitors (ACEIs) or angiotensin II receptor blockers (ARBs) have a renoprotective effect in patients with advanced CKD [3], and for the treatment of hypertension in CKD patients with proteinuria, an ACEI or ARB is the first-line choice [4]. Sodium-glucose cotransporter (SGLT) 2 inhibitors are reported to reduce the incidence of progression to macroalbuminuria and showed surprisingly favorable effects on kidney and cardiovascular outcomes in CKD patients with type 2 diabetes [5], which was recently recognized regardless of the presence of diabetes [6]. Furthermore, addition of an mineralocorticoid receptor (MR) blocker to an ACEI or ARB reduces the urinary albumin-to-creatinine ratio (UACR) in patients with type 2 diabetes [7, 8] and reduces the risk of CKD progression [8]. Moreover, coadministration of an SGLT2 inhibitor and the MR blocker eplerenone has recently demonstrated an additive albuminuria-lowering effect in CKD patients [9]. Although the mechanisms of action may not be the same and are not well known, SGLT2 inhibitors and MR blockers have the potential to reduce urinary protein excretion and delay the progression to ESRD in CKD patients. 

Proteinuria is also an independent predictor of ESRD in kidney transplant recipients [10]. In allograft CKD patients with progressive proteinuria, we administered SGLT2 inhibitors and a nonsteroidal MR blocker, esaxerenone, as an add-on therapy to valsartan and investigated the effect of these agents on urinary protein excretion and kidney function. 

## Case report 

**Figure 1:**
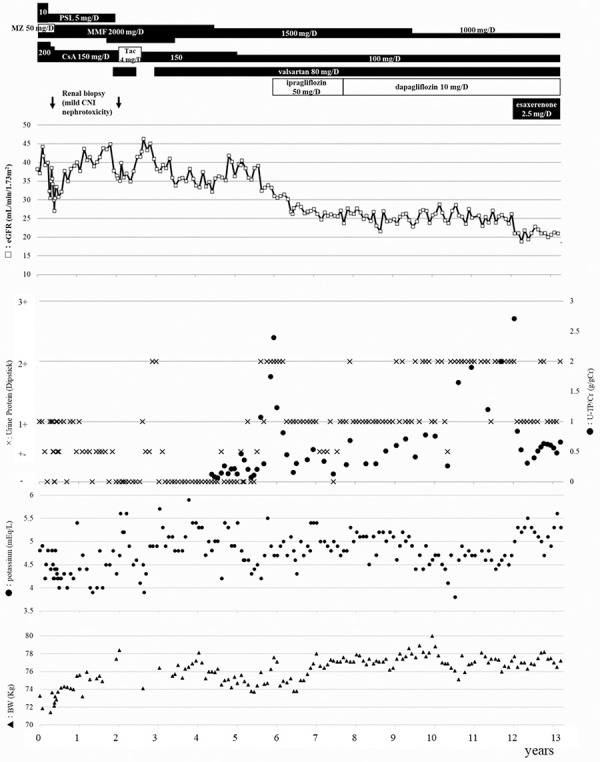
Clinical course of the patient. PSL = prednisolone; MZ = mizoribine; MMF = mycophenolate mofetil; CsA = cyclosporine; Tac = tacrolimus; CNI = calcineurin inhibitor; eGFR = estimated glomerular filtration rate; U-TP/Cr = urinary protein-to-creatinine ratio; BW = body weight; D = day.

A 60-year-old man with presumed chronic glomerulonephritis (i.e., renal biopsy was not performed) underwent a living-donor ABO-compatible kidney transplantation after 4 years of hemodialysis. Initial immunosuppressive medications included prednisolone, mizoribine, mycophenolate mofetil, and cyclosporine. Although the patient had a history of percutaneous coronary intervention, his clinical course was fairly good during the first few years after kidney transplantation. Although posttransplant diabetes mellitus occurred and insulin injection was necessary for 5 years, hemoglobin A1c was controlled at ~ 6% except during the first year after kidney transplantation. Renal graft biopsies were performed at 6 and 26 months after kidney transplantation, both of which revealed mild calcineurin inhibitor nephrotoxicity, and diabetic glomerulopathy was not observed. After the second renal biopsy, cyclosporine was switched to tacrolimus to attempt to mitigate calcineurin inhibitor nephrotoxicity and was again switched to cyclosporine because of hair loss. Two years after kidney transplantation, 80 mg per day of valsartan was administered to treat proteinuria, followed by the disappearance of proteinuria by dipstick with mild increases in serum creatinine and potassium levels. During the 7 months of valsartan discontinuation, proteinuria reappeared, and the serum creatinine and potassium levels returned to the levels before valsartan administration (Figure 1). 

**Figure 2:**
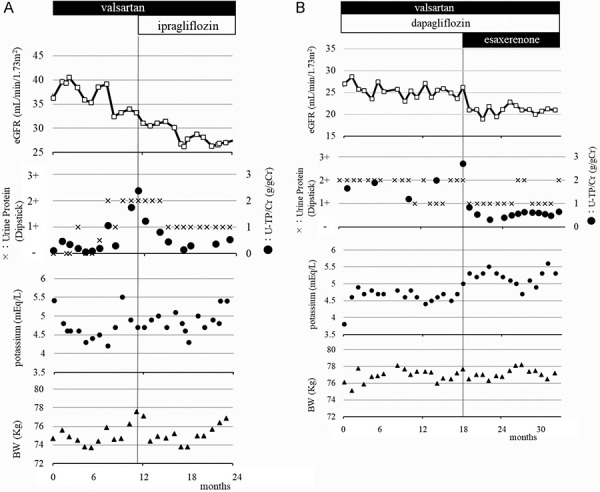
Clinical course of the patient. A: Before and during the administration of ipragliflozin. B: Before and during the administration of esaxerenone. eGFR = estimated glomerular filtration rate; U-TP/Cr = urinary protein-to-creatinine ratio; BW = body weight.

Although kidney function was stable (serum creatinine levels: 1.4 – 1.6 mg/dL), 5 years after kidney transplantation, the urinary protein-to-creatinine ratio (UPCR) increased gradually and was 2.395 g/gCr at 73 months after kidney transplantation. The dipeptidyl peptidase-4 inhibitor sitagliptin for the treatment of posttransplant diabetes mellitus was changed to the SGLT2 inhibitor ipragliflozin to attempt to reduce urinary protein excretion. After the administration of ipragliflozin, urinary protein excretion decreased rapidly, and 3 months later, the UPCR decreased to 0.453 g/gCr with 2.85 kg of body weight loss. After 19 months of ipragliflozin administration, the physician decided to switch from 50 mg per day ipragliflozin to 10 mg per day dapagliflozin because of its more potent cardioprotective effect, since the patient had a history of percutaneous coronary intervention, and the left ventricular ejection fraction was 40%. During the 4 years of SGLT2 inhibitor administration, the UPCR was ~ 0.5 g/gCr, and the serum creatinine levels were ~ 2.0 mg/dL (Figure 1, Figure 2A). 

However, the UPCR increased again to 2.713 g/gCr at 146 months after kidney transplantation. Esaxerenone was administered to try to reduce urinary protein excretion, which was followed by a steep decrease in the UPCR; it was 0.313 g/gCr 3 months later and was maintained at ~ 0.5 g/gCr (Figure 1, Figure 2B). 

During the treatment period of this patient, antihypertensive medications besides valsartan and esaxerenone included amlodipine; the dose was adjusted to between 0 and 10 mg per day, and blood pressure was well controlled at 120 – 140/60 – 80 mmHg. 

## Discussion 

Proteinuria in CKD patients is an independent predictor of ESRD [1]. Because of the renoprotective effect of renin-angiotensin-aldosterone system (RAAS) blockade [3], the use of ACEIs or ARBs is recommended as a first-line treatment in proteinuric CKD patients with hypertension [4]. It is believed that the main mechanism of the decrease in urinary protein excretion and renoprotection is the change in intraglomerular hemodynamics resulting from dilating efferent arterioles. Additionally, it has been reported that candesartan improves size-specific glomerular permselectivity in allograft kidneys [11], which may be the other mechanism by which ACEIs or ARBs decrease urinary protein excretion. In the current case, during the first 4 years after kidney transplantation, urinary protein was negative by dipstick when valsartan was administered. However, urinary protein excretion increased gradually thereafter. 

The etiology of proteinuria in allograft kidneys may not be the same as that in native kidneys. It has been reported that 58% of transplant patients with proteinuria (≥ 150 mg/day) had transplant-specific lesions (allograft nephropathy, transplant glomerulopathy, or acute rejection) on biopsy compared with only 11% with glomerulonephritis [12]. In the current case, the increase in urinary protein excretion 5 years after kidney transplantation may be multifactorial. Although transplant-specific lesions are the possible cause, we have no information on this issue because we did not perform any renal graft biopsies 2 years after kidney transplantation. Additionally, aldosterone escape, which can occur following the long-term administration of valsartan [13], may have augmented proteinuria. 

Administration of the SGLT2 inhibitor ipragliflozin drastically reduced urinary protein excretion in the present case. The UPCR had been maintained at less than 1.0 g/gCr for 4 years by SGLT2 inhibitors. Although the mechanisms by which SGLT2 inhibitors decrease urinary protein excretion and exert their strong renoprotective effects in patients with and without diabetes are not fully understood [14, 15], a reduction in the intraglomerular pressure by glycosuria and natriuresis is one of the possible mechanisms [15], and the acute body weight loss after the initiation of SGLT2 inhibitors in the current case supports this mechanism. 

Esaxerenone is a novel selective MR blocker with a nonsteroidal structure which has 4- or 76-fold higher binding affinity for MR than spironolactone and eplerenone [16]. It has been reported that esaxerenone improves albuminuria in patients with type 2 diabetes as an add-on therapy to ACEIs or ARBs [7]. Finerenone is also a nonsteroidal, selective MR blocker that is reported to reduce the UACR and lower the risk of CKD progression and cardiovascular events in CKD patients with type 2 diabetes [8]. In the current case, the addition of esaxerenone to valsartan and an SGLT2 inhibitor caused a rapid decrease in the UPCR. During the administration of esaxerenone, the serum potassium level and UPCR changed in opposite directions, suggesting the influence of MR blockage by esaxerenone on natriuresis and the reduction in urinary protein excretion. In addition to angiotensin II, aldosterone has a vasoconstrictive effect on glomerular arterioles with a higher sensitivity in efferent than afferent arterioles [17], which can accelerate proteinuria. While it has been reported that the vasoconstrictive effect of aldosterone on glomerular arterioles may be mediated by specific membrane receptors that are distinct from the intracellular MR (nongenomic) and it is not affected by spironolactone, it has also been reported that aldosterone may exert genomic vascular actions [17]; thus, the mechanisms of the glomerular vascular actions of aldosterone are nonconclusive. Although the “initial drop” in the estimated glomerular filtration rate just after the initiation of esaxerenone in the current case suggests the possibility of a decrease in intraglomerular pressure, further study is needed to address this issue. Finally, aldosterone injures podocytes and induces proteinuria [18], and it is possible that esaxerenone exerts an anti-proteinuric effect by ameliorating podocyte injury. 

In CKD patients, SGLT2 inhibitors have cardioprotective and renoprotective effects [6]. Additionally, long-term administration of esaxerenone has the potential to exert renoprotective effects, the mechanisms of which include reduction of urinary albumin excretion [7, 8], and suppression of inflammation and subsequent reduction of renal oxidative stress [19]. Recently, it was reported that coadministration of an SGLT2 inhibitor and eplerenone has demonstrated additive albuminuria-lowering effects in CKD patients [9], and coadministration of SGLT2 inhibitors and MR blockers decreased the risk of hyperkalemia [9, 20], which implies that the coadministration of both agents for CKD patients is safer and may be more renoprotective than SGLT2 inhibitors or MR blockers alone. In the current case, which was a patient with chronic allograft kidney disease, a prompt reduction in urinary protein excretion was achieved safely without any apparent hyperkalemia by add-on therapy of SGLT2 inhibitors and esaxerenone to valsartan. However, the lack of renal biopsy at the time of increasing proteinuria makes it difficult to generalize the results of the current case to the general population. Obtaining evidence for the renoprotective effect of these combination therapies in the general population, as well as in allograft CKD patients, is warranted. 

## Conclusion 

Although the long-term renoprotective effect of the reduction in urinary protein excretion is not known, given that proteinuria in CKD patients is an independent predictor of ESRD [1], it is possible that SGLT2 inhibitors and esaxerenone may be promising and safe agents to induce long-term renoprotection in allograft CKD patients. 

## Acknowledgment 

The author thanks all staff members working at the Daimon Clinic for Internal Medicine, Nephrology. 

## Funding 

No funding was obtained for this study. 

## Conflict of interest 

The author declares that there is no competing interest.
